# Supplementary material for: Multi-View Learning to Unravel the Different Levels Underlying Hepatitis B Vaccine Response
Source: Vaccines (Basel). 2023 Jul 13;11(7):1236. doi: 10.3390/vaccines11071236 (PMC10384938; doi:10.3390/vaccines11071236)
Supplement: Supplementary file 1 [file vaccines-11-01236-s001.zip › Table S1.pdf]

Table S1. **Comparison table of the baseline non-genetic attributes between early and late converters**.

Dataset	Feature	Early-converters (n=21) average (range)	Late-converters (n=9) average (range)
Metadata	Age (years)	35.9 (21.3-50.2)	44.19 (36.3-48.5)
	Temperature ([degree]C)	36.60 (35.92-37.25)	36.57 (35.35-37.19)
	Max BP (mmHg)	126.90 (98-148)	122.11 (103-157)
	Min BP (mmHg)	75.62 (55-93)	74.67 (59-105)
	Female/Male	13/8*	7/2*
CD4+ T cell parameters	B0 (counts)	50790.38 (37866-71140)	50237.89 (29330-87551)
	PPnrB0 (%)	2.71 (2.01-3.45)	3.37 (1.72-5.62)
	PSB0 (%)	2.75 (1.87-3.87)	2.49 (1.40%-4.53%)
	HepBTCRs (counts)	1.02 (0.74-1.36)	0.78 (0.42-1.11)
Cell counts	WBC0 (counts)	6.10 (3.6-9.4)	6.74 (4.3-9.3)
	RBC0 (counts)	4.59 (3.94-5.21)	4.60 (4.14-5.42)
	HGB0 (counts)	13.62 (11.5-15.5)	13.30 (8.1-15.4)
	HCT0 (counts)	40.67 (35.0-46.4)	39.82 (26.2-46.2)
	PLT0 (counts)	243.67 (144.0-351.0)	242.89 (172.0-403.0)
	LYM0 (counts)	34.14 (19.5-47.8)	29.7 (15.3-38.7)
	MON0 (counts)	4.79 (3.2-8.2)	4.13 (2.5-7.4)
	GRA0 (counts)	61.07 (49.0-76.6)	66.17 (57.4-77.3)

See the original manuscripts [19], [24] for breakdown of features.

Maximum blood pressure (day 0) (Max BP); minimum blood pressure (day 0) (Min BP); total unique TCR sequences (day 0) (B0); fraction of HBs-Ag-aspecific (day 0) (PPnrB0); fraction of HBs-Ag-specific (day 0) (PSB0); normalised ratio of vaccine-specific TCRs (day 0) (HepBTCRs); white blood cells count (day 0) (WBCO); red blood cells count (day 0) (RBC0); haemoglobin protein count (day 0) (HGB0); hematocrit (day 0) (HCT0); platelets count (day 0) (PLT0); lymphocytes count (day 0) (LYM0); monocytes count (day 0) (MON0); granulocytes count (day 0) (GRA0). *Number of individuals.

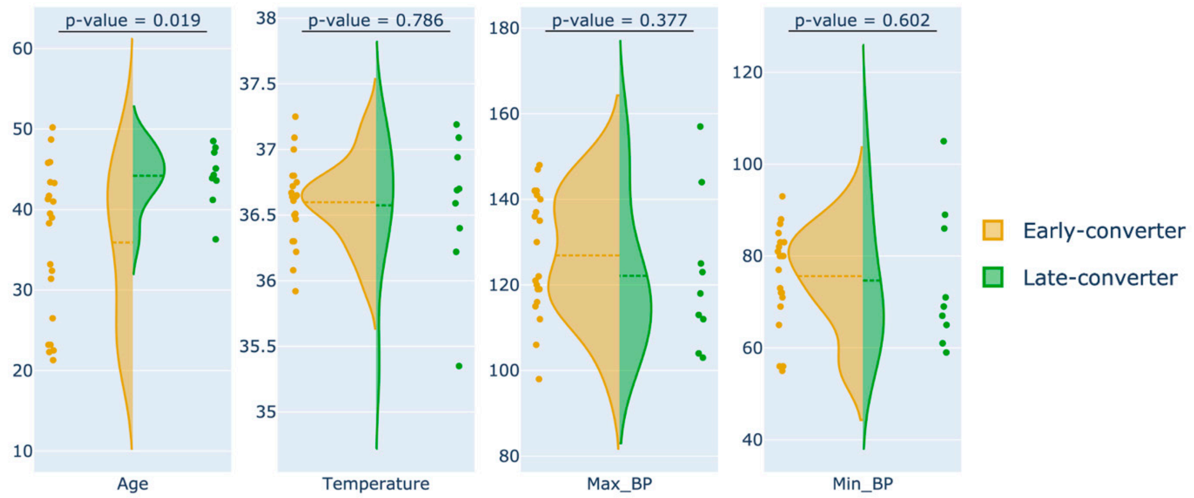

Figure S1. Metadata distributions reveal age differences. Metadata feature distributions per class. Age is significantly higher for late converters (p-value = 0.019, two-sided Mann-Whitney U-test).

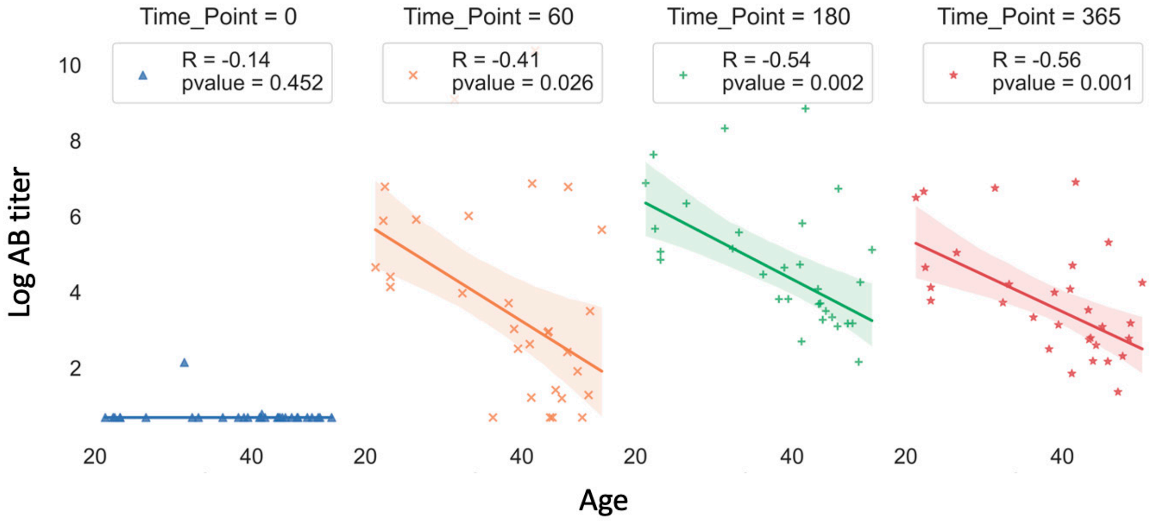

Figure S2. Longitudinal analysis of AB titers shows anticorrelation with age. Comparison between age and AB for early and late converters, presented at each timepoint of the study. Time is expressed in days from the first vaccine shot. Antibody (AB).

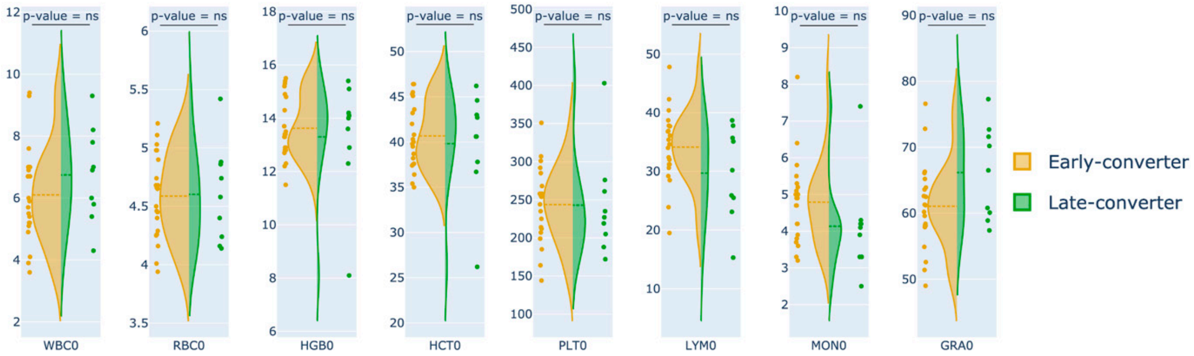

Figure S3. Cell counts distributions reveal class differences. Cell counts feature distributions per class. Granulocytes and monocytes appear in lower quantities, respectively, for early and late converters. However, possible outliers lead to non-significant differences.

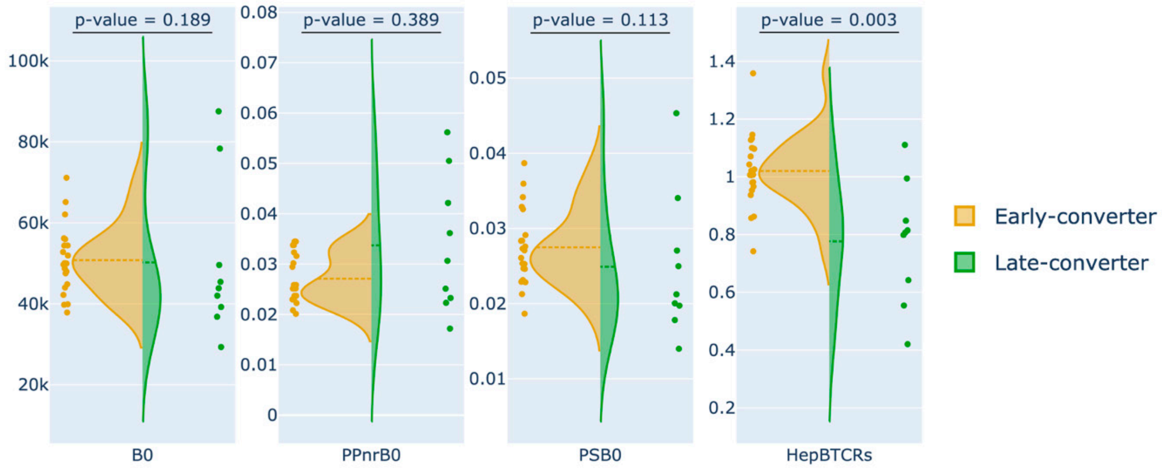

Figure S4. CD4+ T cell parameters reveal class differences. Pre-vaccination TCR data feature distribution per class. HepBTCRs are significantly elevated in early vaccine responses (p-value = 0.003, two-sided Mann-Whitney U-test). T-Cell Receptor (TCR).

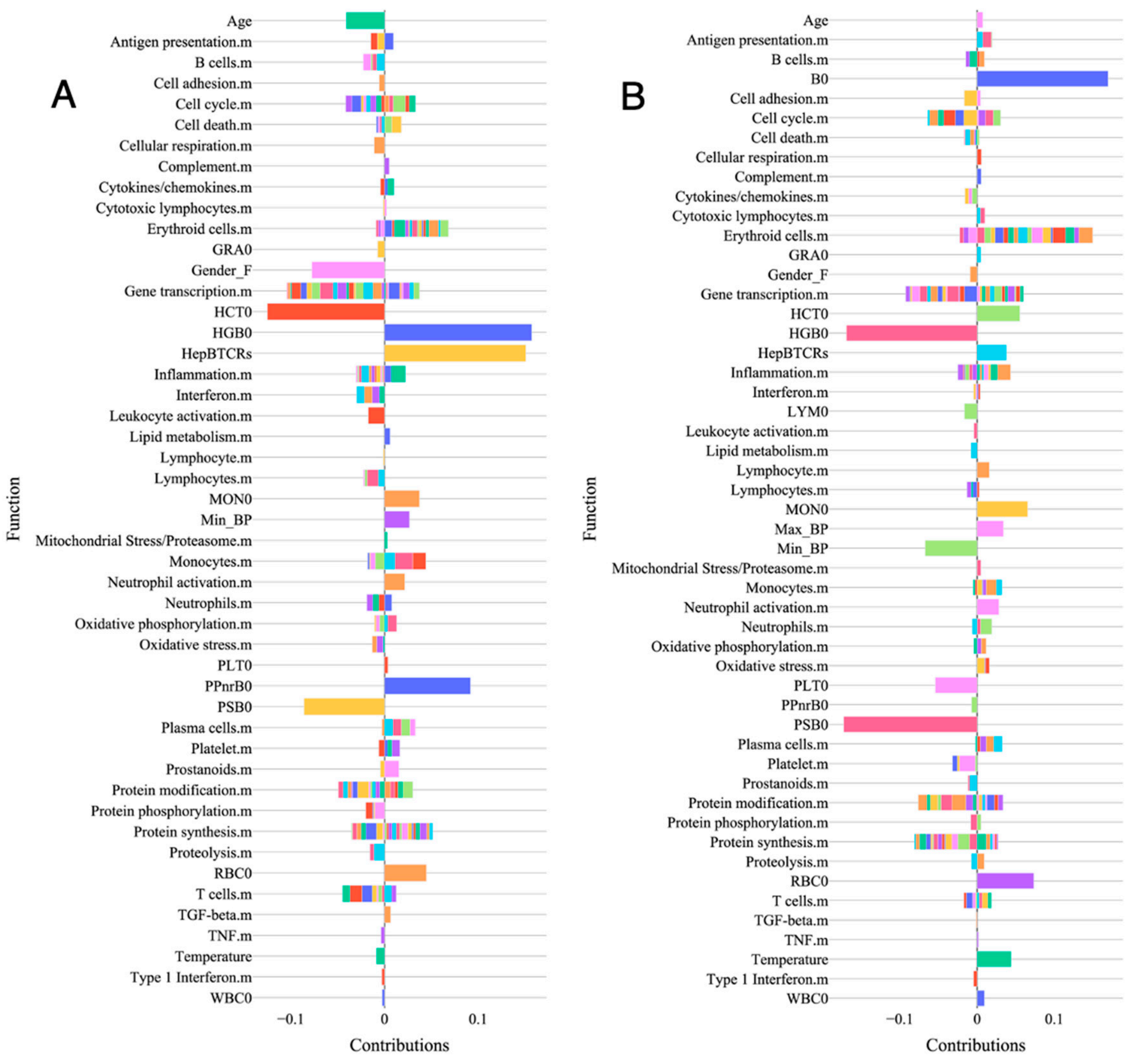

Figure S5. MCCA reveals distinct contributions of the metadata and expression. Visualisations of the loadings to the first (A) and second (B) dimensions of the MCCA projection. Coefficients were stacked by function, with null-values and modules without associated functionality being omitted. Multi-view Canonical Correlation Analysis (MCCA).

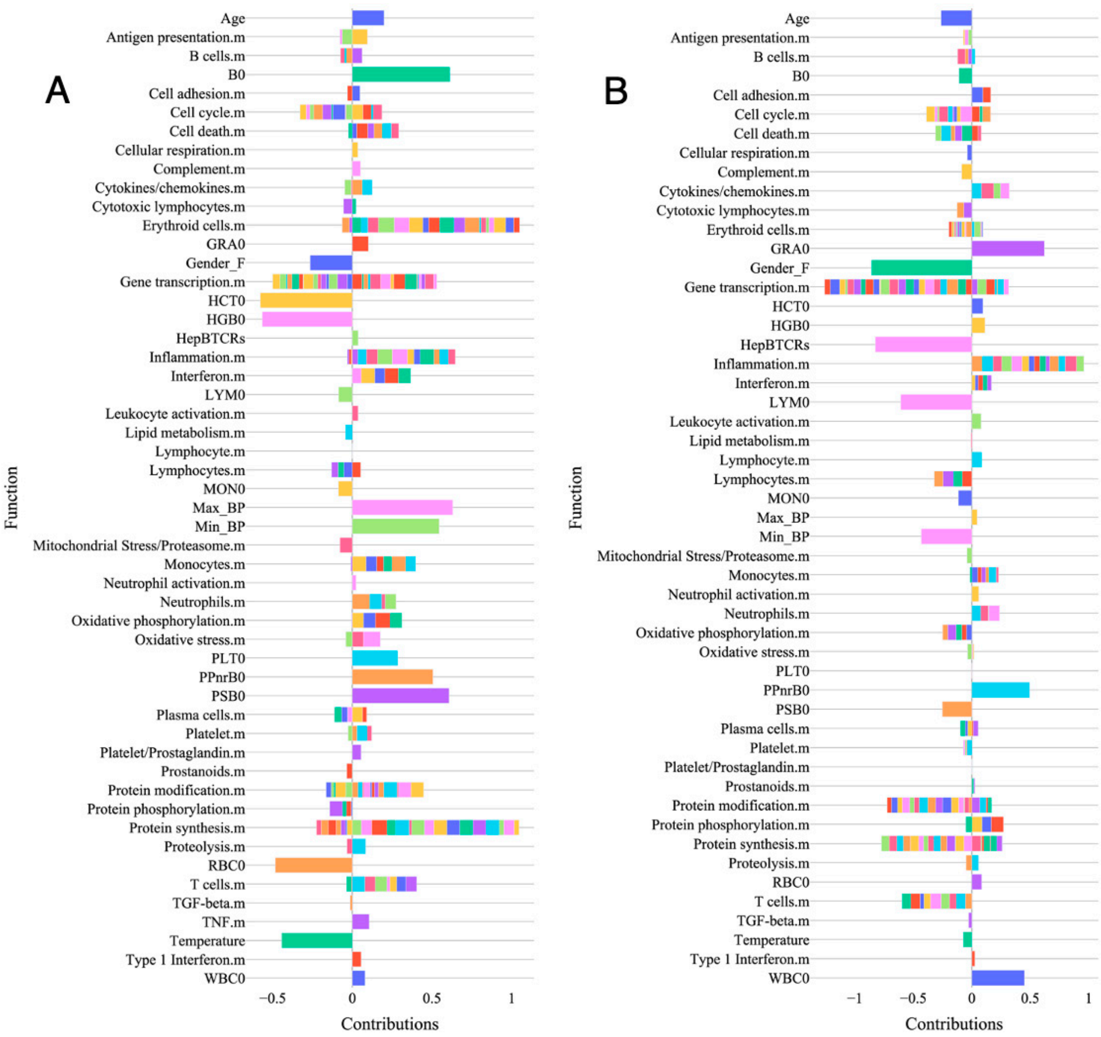

Figure S6. GroupPCA components support MCCA findings and offer better interpretability. Visualisations of the loadings to the first (A) and second (B) dimensions of the GroupPCA projection. Coefficients were stacked by function, with null-values and modules without associated functionality being omitted. Principal Component Analysis (PCA).

Table S2. Performance table for the evaluation in terms of AUC and accuracy of a LR trained on single modality data.

View	Cell counts	TCR-seq	Metadata	RNA-seq
Accuracy	0.506±0.044	0.708±0.067	0.598±0.058	0.440±0.018
AUC	0.430±0.038	0.731±0.040	0.588±0.028	0.611±0.009

Area Under Curve (AUC); Logistic Regression (LR).

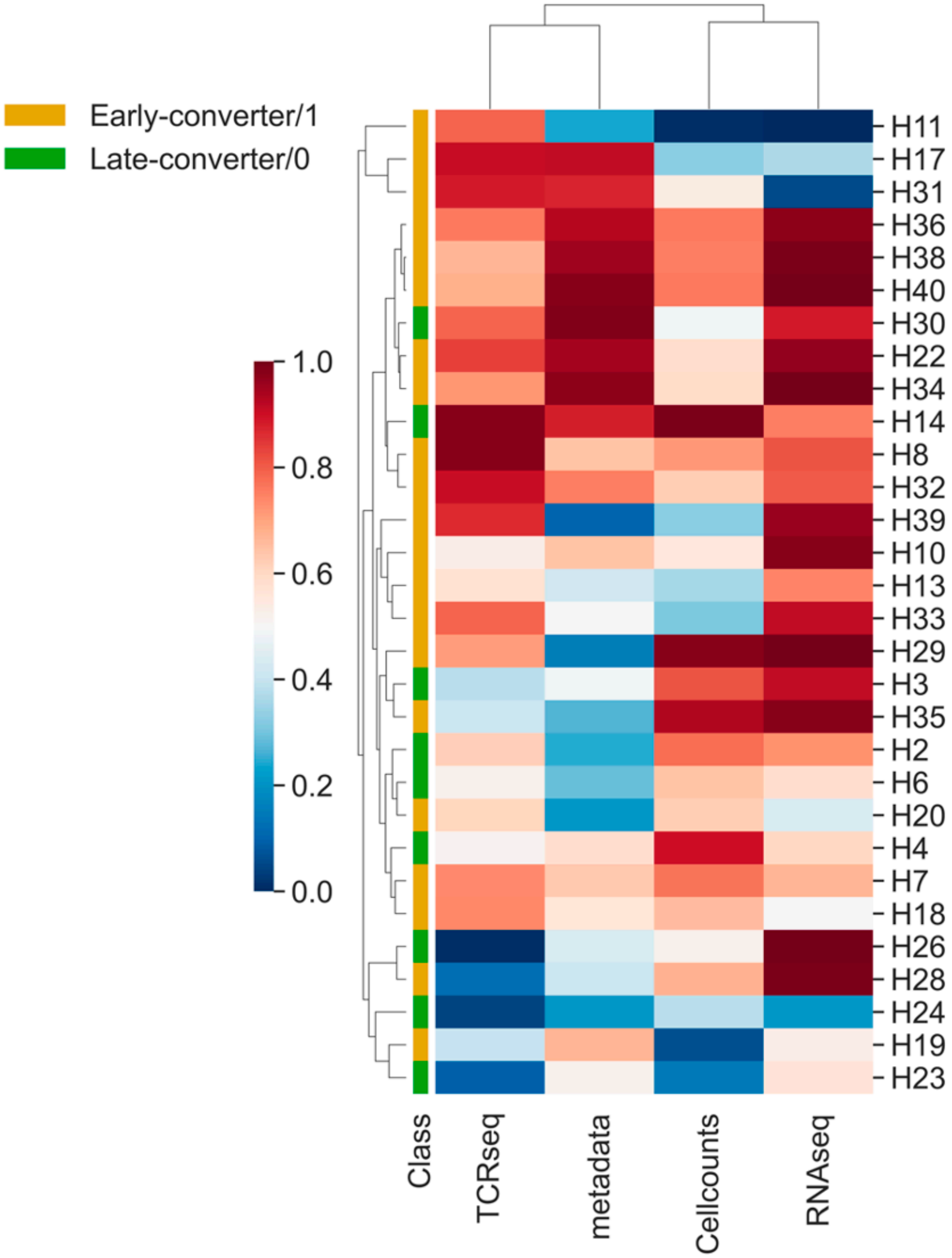

Figure S7. Unimodal predicted probabilities show layer to layer difference in predictive power. Predicted probabilities attributed to each patient of the cohort, per single data modality of a LOOCV LR. Leave-One-Out Cross-Validation (LOOCV).
