# Supplementary figures and images for: Multi-View Learning to Unravel the Different Levels Underlying Hepatitis B Vaccine Response
Source: Vaccines (Basel). 2023 Jul 13;11(7):1236. doi: 10.3390/vaccines11071236 (PMC10384938; doi:10.3390/vaccines11071236)

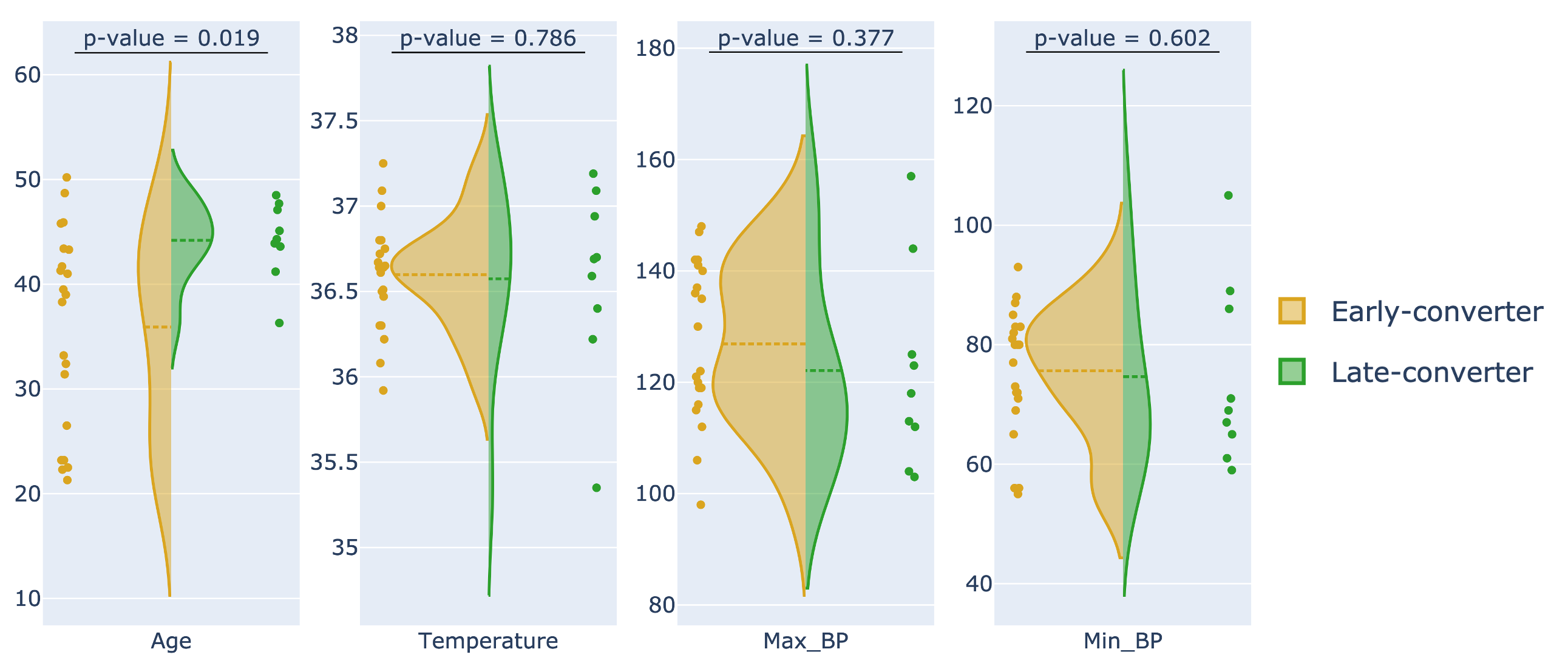

Supplement: Supplementary file 1 [file vaccines-11-01236-s001.zip › Figure S1.png]

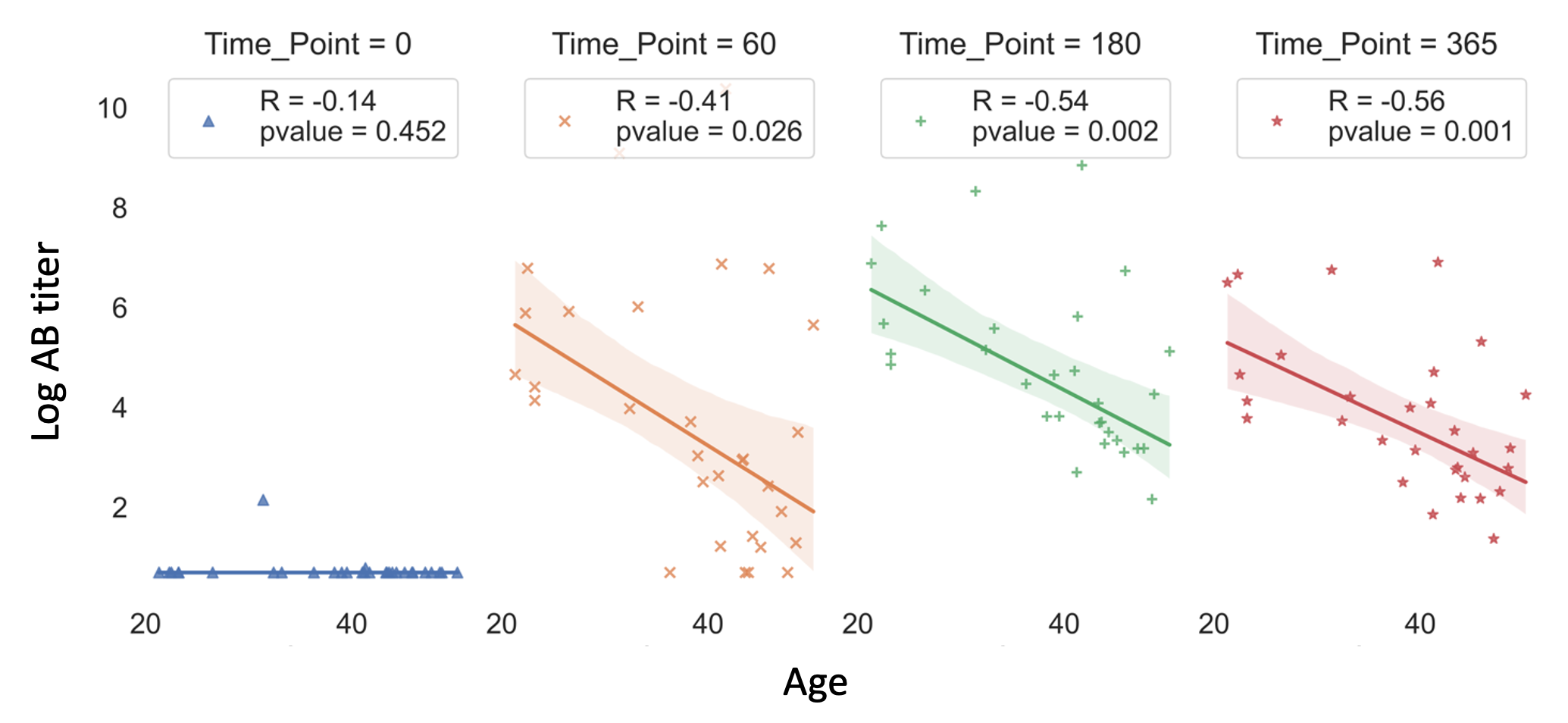

Supplement: Supplementary file 1 [file vaccines-11-01236-s001.zip › Figure S2.png]

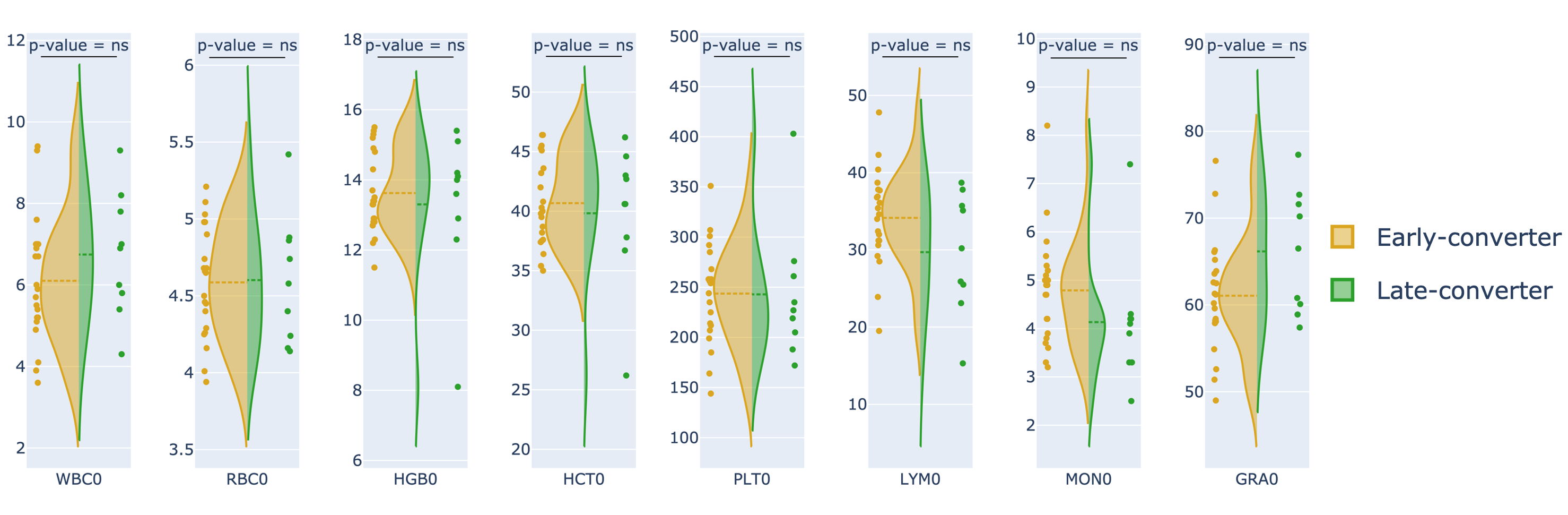

Supplement: Supplementary file 1 [file vaccines-11-01236-s001.zip › Figure S3.png]

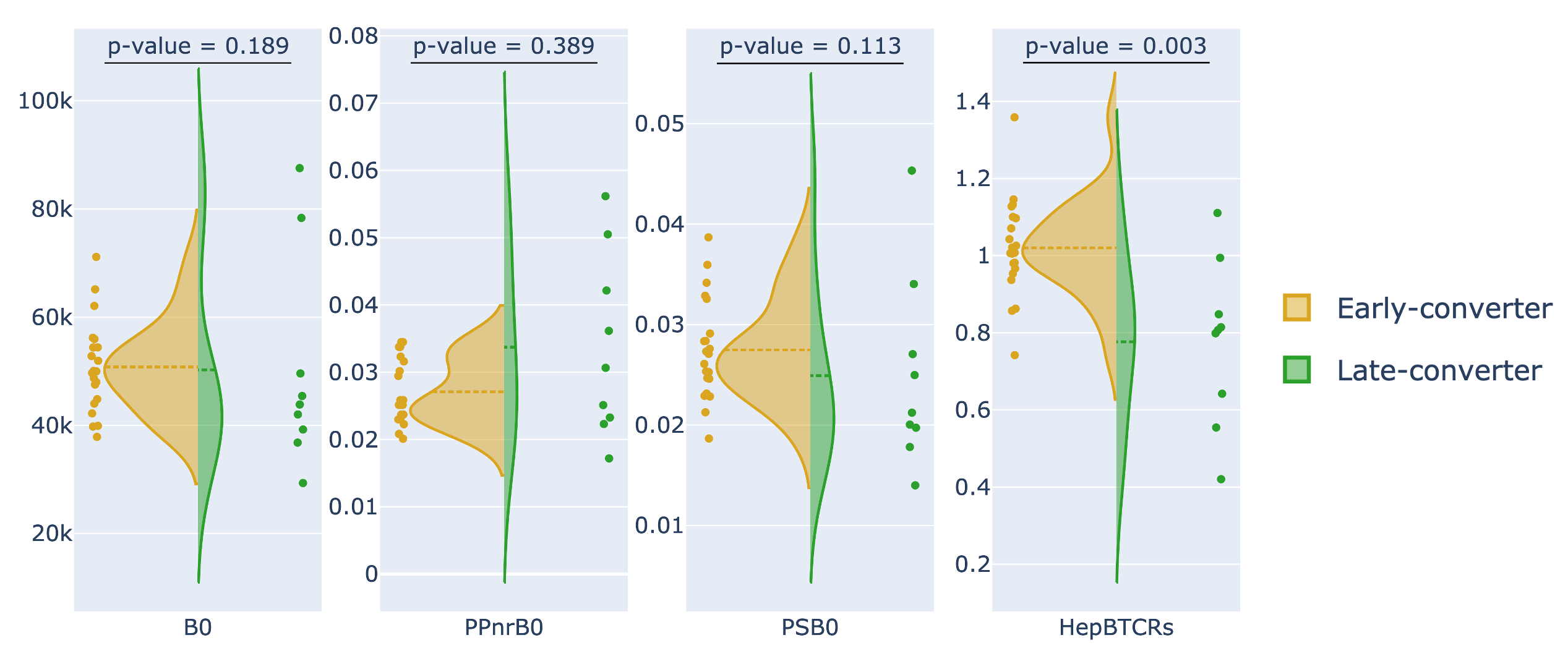

Supplement: Supplementary file 1 [file vaccines-11-01236-s001.zip › Figure S4.png]

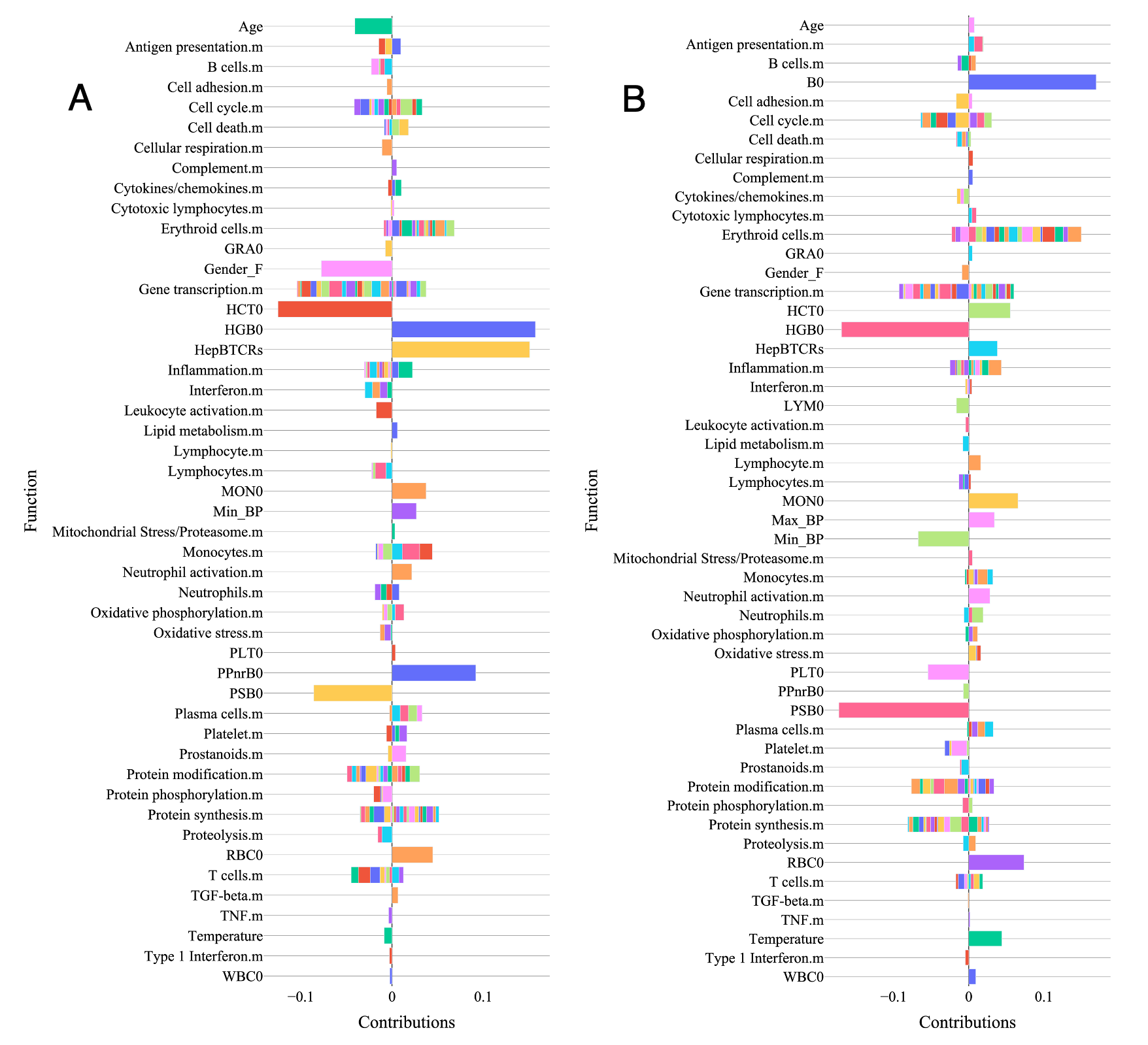

Supplement: Supplementary file 1 [file vaccines-11-01236-s001.zip › Figure S5.png]

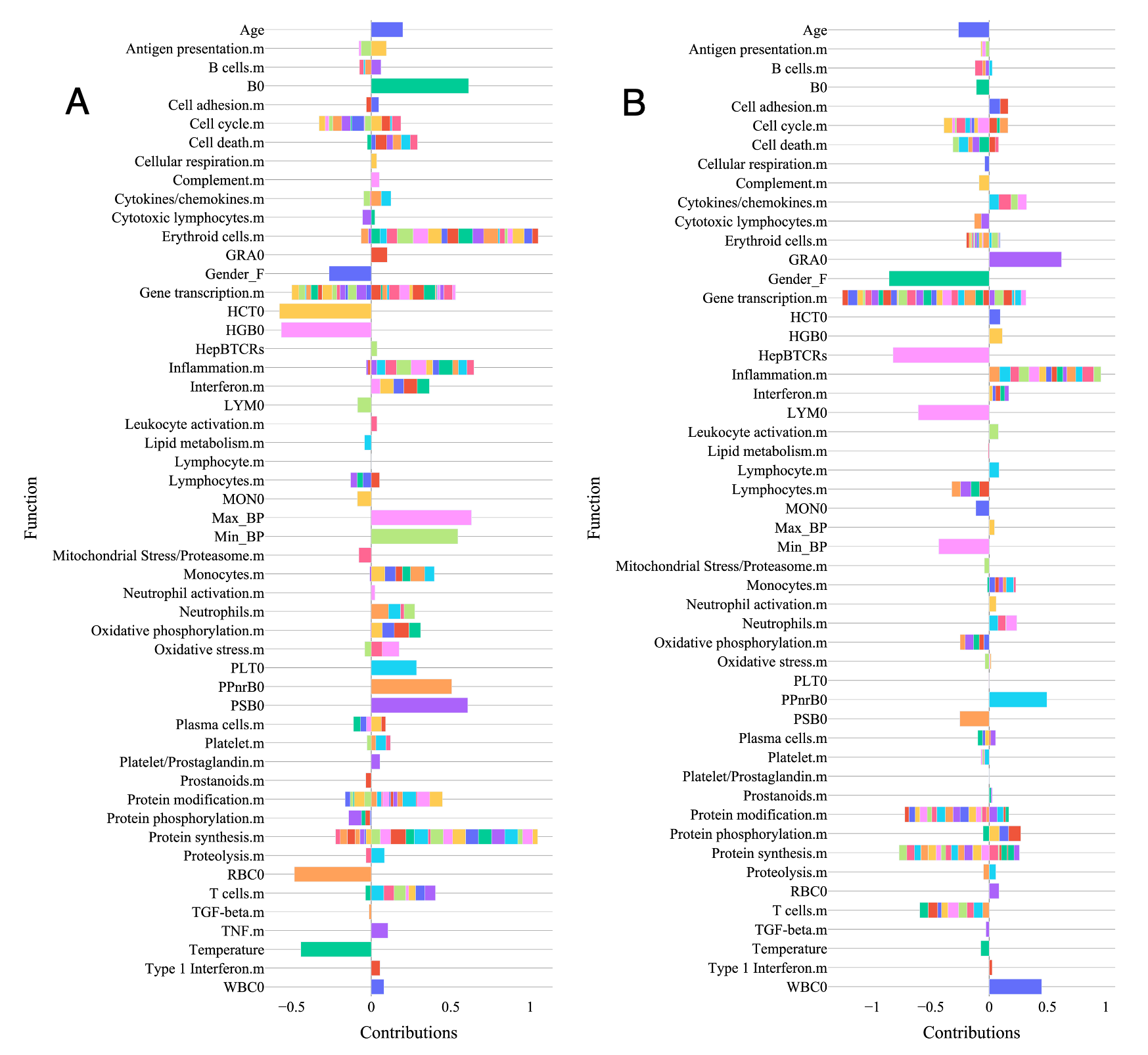

Supplement: Supplementary file 1 [file vaccines-11-01236-s001.zip › Figure S6.png]

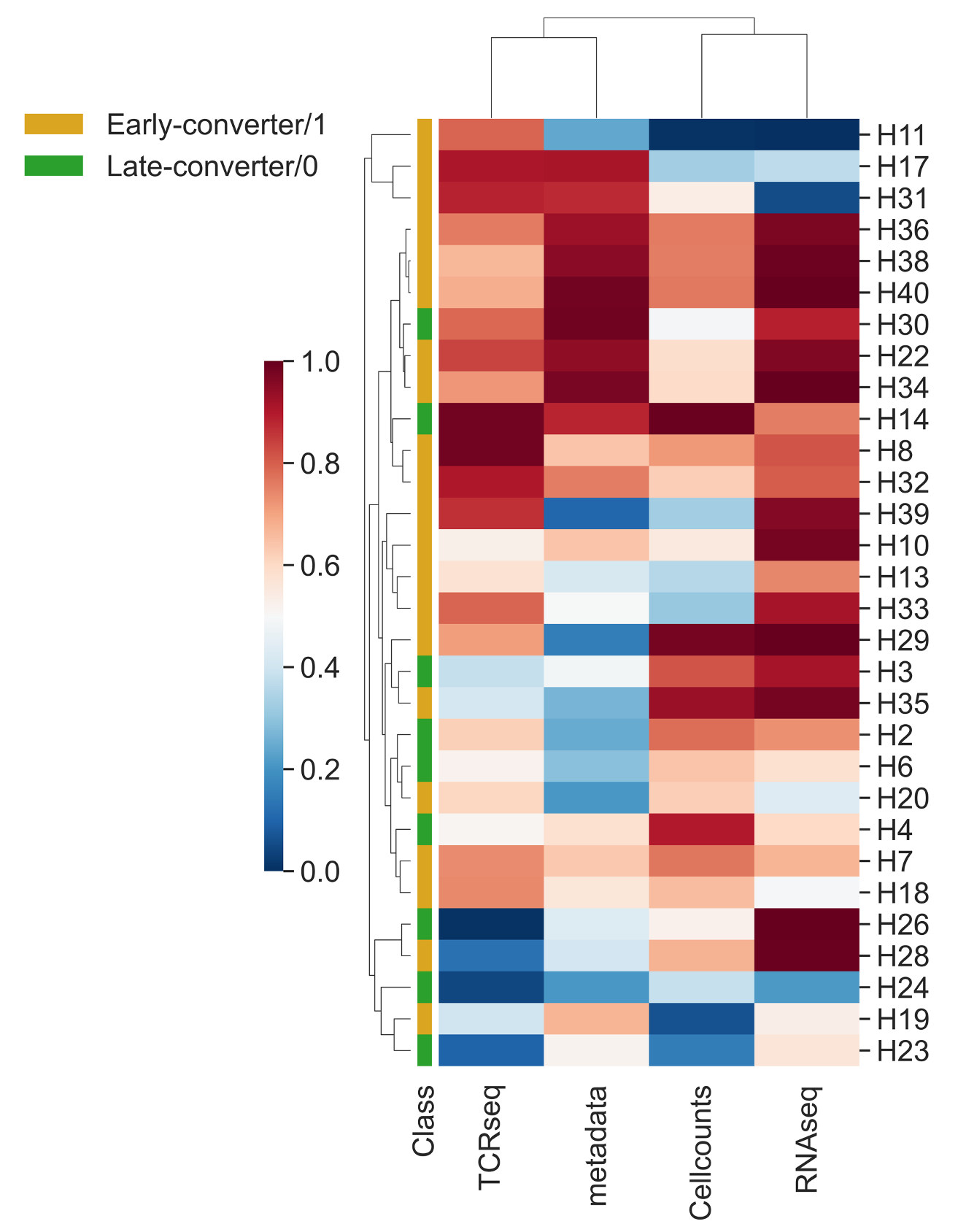

Supplement: Supplementary file 1 [file vaccines-11-01236-s001.zip › Figure S7.png]
